# Pd_2_MnGa Metamagnetic Shape Memory Alloy with Small Energy Loss

**DOI:** 10.1002/advs.202207779

**Published:** 2023-06-12

**Authors:** Tatsuya Ito, Xiao Xu, Atsushi Miyake, Yuto Kinoshita, Makoto Nagasako, Kohki Takahashi, Toshihiro Omori, Masashi Tokunaga, Ryosuke Kainuma

**Affiliations:** ^1^ Department of Materials Science Graduate School of Engineering Tohoku University Aoba‐yama 6‐6‐02 Sendai 980‐8579 Japan; ^2^ Organization for Advanced Studies Tohoku University Katahira 2‐1‐1 Sendai 980‐8577 Japan; ^3^ The Institute for Solid State Physics The University of Tokyo Kashiwanoha 5‐1‐5 Kashiwa 277‐8581 Japan; ^4^ Institute for Materials Research Tohoku University Katahira 2‐1‐1 Sendai 980‐8577 Japan; ^5^ Present address: J‐PARC Center Japan Atomic Energy Agency Shirakata 2‐4 Tokai 319‐1195 Japan; ^6^ Present address: Institute for Materials Research Tohoku University Katahira 2‐1‐1 Sendai 980‐8577 Japan

**Keywords:** Heusler alloys, martensitic transformation, martensitic transformation hysteresis, metamagnetic shape memory alloys, Pd_2_MnGa alloy

## Abstract

Metamagnetic shape memory alloys (MMSMAs) are attractive functional materials owing to their unique properties such as magnetostrain, magnetoresistance, and the magnetocaloric effect caused by magnetic‐field‐induced transitions. However, the energy loss during the martensitic transformation, that is, the dissipation energy, *E*_dis_, is sometimes large for these alloys, which limits their applications. In this paper, a new Pd_2_MnGa Heusler‐type MMSMA with an extremely small *E*_dis_ and hysteresis is reported. The microstructures, crystal structures, magnetic properties, martensitic transformations, and magnetic‐field‐induced strain of aged Pd_2_MnGa alloys are investigated. A martensitic transformation from *L*2_1_ to 10*M* structures is seen at 127.4 K with a small thermal hysteresis of 1.3 K. The reverse martensitic transformation is induced by applying a magnetic field with a small *E*_dis_ (= 0.3 J mol^−1^ only) and a small magnetic‐field hysteresis (= 7 kOe) at 120 K. The low values of *E*_dis_ and the hysteresis may be attributed to good lattice compatibility in the martensitic transformation. A large magnetic‐field‐induced strain of 0.26% is recorded, indicating the proposed MMSMA's potential as an actuator. The Pd_2_MnGa alloy with low values of *E*_dis_ and hysteresis may enable new possibilities for high‐efficiency MMSMAs.

## Graphical abstract

Metamagnetic shape memory alloys (MMSMAs) are attracting attention as multifunctional materials, such as magnetic actuators and environment‐friendly magnetorefrigeration materials. However, the large energy loss during the phase transformation hinders their applications. In this study, a novel Pd_2_MnGa MMSMA with extremely small energy loss, which may lead to high efficiency, has been developed.

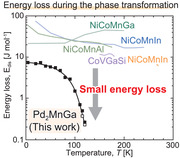

## 1 Introduction

Metamagnetic shape memory alloys (MMSMAs), which exhibit reversible martensitic transformations when a magnetic field is applied, have recently attracted attention as multifunctional materials.^[^ ^1^, ^2^ ^]^ One of the expected applications is their use as magnetic actuators. A large output strain of more than 5% can be obtained through the magnetic‐field‐induced reverse martensitic transformation in Ni–Co–Mn–In alloys. In addition, a strong output stress can be obtained owing to the large driving force caused by the difference in Zeeman energy between the parent and martensite phases.^[^ ^3^, ^4^, ^5^ ^]^ The energy density of MMSMAs is estimated to be higher than those of other known magnetostrictive alloys, such as Terfenol‐D.^[^ ^4^ ^]^ Moreover, solid‐state refrigeration techniques are highly efficient and, thus, appropriate for the realization of a sustainable society.^[^ ^6^, ^7^ ^]^ For example, MMSMAs are expected to be applied as magnetorefrigeration materials that utilize the magnetocaloric effect,^[^ ^8^, ^9^ ^]^ where the large entropy change during the martensitic transformation is used. Several alloy systems have been developed for MMSMAs, among which Ni–Mn‐based Heusler alloys have been widely investigated.^[^ ^10^, ^11^, ^12^ ^]^

Energy dissipation and hysteresis during phase transformation are critical obstacles that should be overcome for the application of MMSMAs. During the operating cycle of MMSMAs, a part of the input energy is wasted as heat owing to the first‐order martensitic transformation; this is referred to as the dissipation energy (*E*_dis_). For applications such as actuators, a lower value of *E*_dis_ is advantageous; it not only leads to lower energy consumption but also causes reduced magnetic‐field hysteresis (Δ*H*_hys_) and temperature hysteresis (Δ*T*_hys_) during operation. On the contrary, a larger hysteresis is favorable for applications such as pipe coupling and prestressed concrete.^[^ ^13^, ^14^ ^]^ Furthermore, an increase in Δ*H*_hys_ has been reported for Ni–Co–Mn–In alloys at low temperatures, which limits the applicable temperature range to approximately room temperature.^[^ ^15^ ^]^ In recent years, applications in low‐temperature environments have increased owing to the demands of the aerospace industry and upcoming hydrogen society. Therefore, there are several potential applications of a new MMSMA with low *E*_dis_ and Δ*H*_hys_ values at low temperatures.

An improvement in the lattice compatibility between the parent and martensite phases effectively reduces hysteresis in martensitic transformations. Cui et al. proposed that λ_2_, which is the middle eigenvalue of the transformation stretch matrix,^[^ ^16^ ^]^ has a significant effect on the thermal hysteresis of Ti–Ni‐based alloys. The value of λ_2_ can be calculated using the knowledge of the crystal structures and lattice constants of the parent and martensite phases.^[^ ^16^, ^17^ ^]^ In addition, λ_2_ depends considerably on the composition of the alloy; this dependence of the hysteresis has been investigated in various Ni–Mn‐based MMSMA systems.^[^ ^18^, ^19^, ^20^, ^21^ ^]^ Therefore, *E*_dis_ was successfully reduced to ≈5 J mol^−1^ at a low value of Δ*H*_hys_ (≈11 kOe) in the room‐temperature range.^[^ ^19^ ^]^ However, Δ*H*_hys_ is still considered large. Some shape memory alloys, such as Ti–Ni and Fe–Pd alloys, exhibit low values of *E*_dis_ (0.3–1.0 J mol^−1^);^[^ ^22^, ^23^ ^]^ however, they cannot be driven by magnetic fields. To the best of our knowledge, such low values of *E*_dis_ have not been achieved for MMSMAs.

Several studies have been conducted on Ni–Mn‐based alloys; however, few studies are available on Pd–Mn‐based alloys, in which Ni is replaced by an element of the same family, Pd. Martensitic transformation has been reported in Mn‐rich Pd–Mn–Sn alloys, and a magnetic phase diagram has been constructed.^[^ ^24^, ^25^, ^26^ ^]^ A magnetic‐field‐induced reverse martensitic transformation has been confirmed, and a large magnetoresistance of −30% has been reported.^[^ ^27^ ^]^ Furthermore, a spin‐flop‐type metamagnetic transition has been reported for Pd_2_MnIn Heusler alloys.^[^ ^28^ ^]^

Our previous studies presented the martensitic transformation and shape memory effect for Pd_2_Mn_2 − *x*_Ga_*x*_ alloys.^[^ ^29^, ^30^ ^]^ A magnetic phase diagram was constructed, and it was reported that the martensitic transformation temperature decreased with the addition of Ga. However, this investigation was limited to high‐temperature heat‐treated alloys with a *B*2 structure. The stoichiometric Pd_2_MnGa alloy with a *B*2 structure is antiferromagnetic with a Néel temperature of 198 K, and it does not undergo martensitic transformation. Furthermore, metamagnetic transitions have not been reported.

In this study, an aged Pd_2_MnGa alloy exhibited a martensitic transformation for the first time and a magnetic‐field‐induced reverse martensitic transformation was also realized. A small hysteresis (Δ*T*_hys_ = 1.3 K and Δ*H*_hys_ = 7 kOe) and significantly low dissipation energy (*E*_dis_ = 0.3 J mol^−1^) are unique characteristics of this martensitic transformation. A magnetic‐field‐induced strain of up to 0.26% was recorded; thus, the newly developed MMSMA is a potential highly efficient actuator.

## 2 Results

### 2.1 Thermoelastic Martensitic Transformation

**Figure 1:**
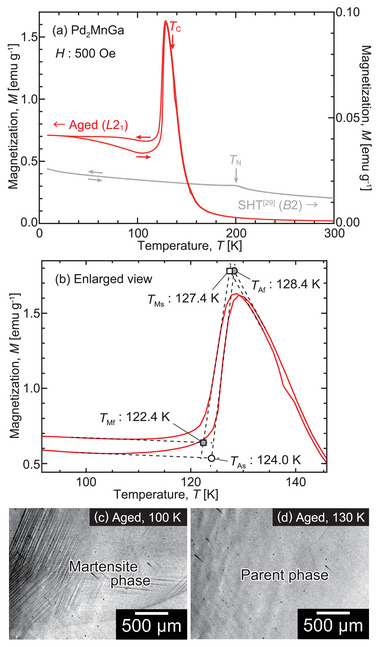
a) Thermomagnetization curve of the aged Pd_2_MnGa alloy at 500 Oe (red line). The reported thermomagnetic response of a solution heat‐treated (SHT) Pd_2_MnGa alloy is also shown as the gray line.^[^ ^29^ ^]^ b) Enlarged view of the onset of the martensitic transformation, where the forward martensitic transformation starting and finishing temperatures (*T*_Ms_, *T*_Mf_) and the reverse martensitic transformation starting and finishing temperatures (*T*_As_, *T*_Af_) are determined using the extrapolation method. c,d) Micrographs obtained through in situ optical microscopic observations at low temperatures. The surface relief at 100 K suggests martensitic transformation, which corresponds to the sharp variation in magnetization displayed in (a,b).

The thermomagnetization curve for the aged Pd_2_MnGa alloy is shown in **Figure** **1**a; the solution heat‐treated (SHT) Pd_2_MnGa alloy with antiferromagnetism reported in a previous study is also depicted.^[^ ^29^ ^]^ It is clear that the magnetization increases upon aging. The parent phase becomes ferromagnetic or ferrimagnetic with a Curie temperature (*T*_C_) of 137 K, which is defined as the temperature with a maximum gradient. A sharp decrease in the magnetization was seen below the Curie temperature, which suggests another phase transformation.

To clarify the origin of this phenomenon, the microstructure was observed using an in situ optical microscope, as shown in Figure 1c,d. Surface relief was observed upon cooling and disappeared upon heating, which is clear evidence of the first observation of the thermoelastic martensitic transformation of the stoichiometric Pd_2_MnGa alloy. The martensitic transformation temperatures were determined using extrapolation methods, as shown in Figure 1b. The martensitic transformation starting temperature (*T*_Ms_) and finishing temperature (*T*_Mf_) were determined as 127.4 and 122.4 K, respectively, whereas the reverse martensitic transformation starting temperature (*T*_As_) and finishing temperature (*T*_Af_) were determined as 124.0 and 128.4 K, respectively. The mean martensitic temperature (*T*_M0_) and austenitic temperature (*T*_A0_) were calculated as $T_{M0}=\left(T_{\mathrm{Ms}}+T_{\mathrm{Mf}}\right)/2=124.9$ K and $T_{A0}=\left(T_{\mathrm{As}}+T_{\mathrm{Af}}\right)/2=126.2$ K. Therefore, an extremely small thermal hysteresis (Δ*T*_hys_ = 1.3 K) was recorded, which is defined as $T_{A0}-T_{M0}$. The transformation temperature width of the forward martensitic transformation, defined as $T_{\mathrm{Ms}}-T_{\mathrm{Mf}}$, was calculated as 5.0 K.

### 2.2 Crystal Structure Determination

**Figure 2:**
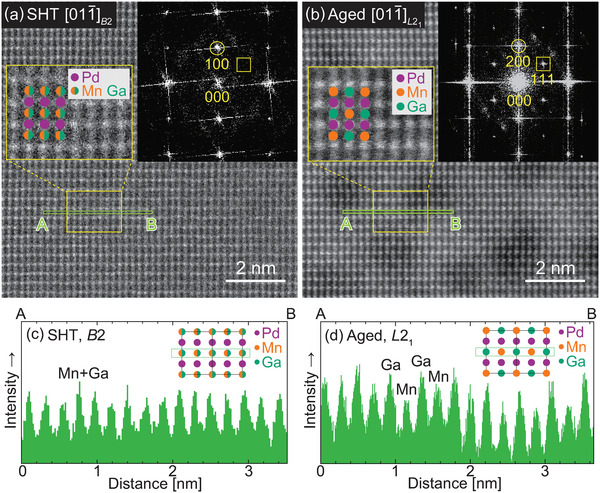
Scanning transmission electron microscopy–high‐angle annular dark‐field (STEM‐HAADF) images of the a) solution heat‐treated (SHT) and b) aged Pd_2_MnGa alloys. The fast Fourier transform (FFT) images are shown as insets. The crystal structure of the SHT sample was determined to be *B*2, while that of the aged sample was *L*2_1_. c,d) Line profiles of the Mn and Ga sites. The intensities of the Mn and Ga sites are almost constant for the *B*2 structure owing to the random occupation of the Mn and Ga atoms. However, there is an alternating stronger and weaker intensity for the *L*2_1_ structure, owing to the ordering of the Mn and Ga atoms.

The crystal structure of the parent phase was investigated using scanning transmission electron microscopy (STEM). The atomic‐resolution high‐angle annular dark‐field–STEM (HAADF‐STEM) images of the SHT and aged Pd_2_MnGa alloys are shown in **Figure** **2**a,b, respectively, and the fast Fourier transform (FFT) images are shown in the insets. The direction of the incident electron beam was parallel to the [01$\bar{1}$] direction of the BCC phase. The columns of the Pd sites are brighter because the atomic number of Pd is considerably larger than those of Mn and Ga. In the FFT images of both the SHT and aged alloys, the superlattice reflection for the *B*2 structure was observed (circled spots); however, the superlattice reflection for the *L*2_1_ structure was observed only in the aged sample (squares). These results indicate that the SHT sample has a *B*2 structure, which is consistent with our previous study;^[^ ^29^ ^]^ further ordering towards the *L*2_1_ structure occurred during the aging heat treatment. The line profiles also support this conclusion for the Mn and Ga sites, as indicated in Figure 2c,d, respectively. The intensities of the line profiles are almost constant for the *B*2 structure because Mn and Ga randomly occupy the Mn and Ga sites. In contrast, zigzag intensities were seen in the profiles when the crystal structure changed to *L*2_1_.

The exchange interactions between Mn atoms depend on the Mn–Mn distances.^[^ ^31^ ^]^ The magnetic interaction changes from antiferromagnetic to ferromagnetic through atomic ordering from *B*2 to *L*2_1_ in Ni–Mn–Al and Ni–Mn–Ga–Al alloys.^[^ ^32^, ^33^ ^]^ The ordering from *B*2 to *L*2_1_ expands the Mn–Mn distances from the second to the third nearest neighbor, which is a possible origin of the increased magnetization after aging for the current Pd_2_MnGa alloy.

In addition, round contrasts ≈2 nm in size appeared in the STEM‐HAADF image of the aged sample, which may be related to nanoscale composition fluctuations caused by the aging heat treatment. However, it was difficult to clarify the origin of the contrasts in this study; therefore, this will be a subject for future research.

**Figure 3:**
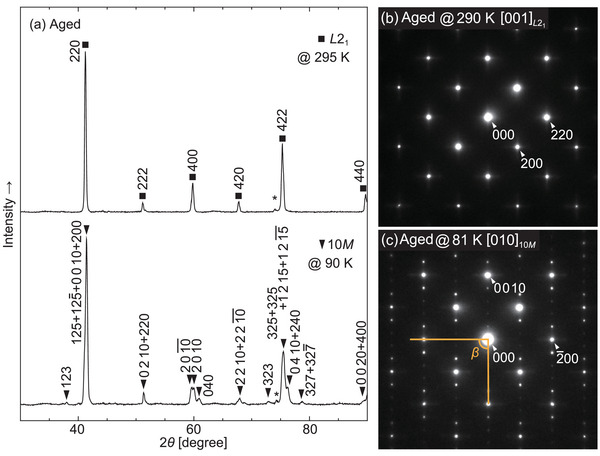
a) In situ powder X‐ray diffraction (XRD) patterns at 295 and 90 K using Cu‐Kα (λ = 1.5418 Å) radiation for the aged Pd_2_MnGa alloy. Unknown peaks are marked with an asterisk (*). The peaks indicate *L*2_1_ and 10*M* structures for the parent and martensite phases, respectively. The peak splittings of 2 0 10 and 2 0 $\bar{10}$ suggest that the martensite phase is monoclinic. b,c) Selected area diffraction patterns of the aged sample measured at 290 K (b) and 81 K (c) by in situ transmission electron microscopy (TEM) observations. Four extra spots indicating a 10*M* structure were found at 81 K. The results of the in situ TEM observations are in good agreement with those of the in situ XRD measurements.

The crystal structures at low temperatures were determined using in situ X‐ray diffraction (XRD) measurements and in situ transmission electron microscopy (TEM) observations. The powder XRD patterns of the aged Pd_2_MnGa alloy at 295 and 90 K are shown in **Figure** **3**a. The XRD pattern at 295 K is consistent with the *L*2_1_ structure. An additional unknown peak, marked with an asterisk (*), is seen, which was also reported for the SHT sample.^[^ ^29^ ^]^ During cooling, the peaks split upon the martensitic transformation.

In situ TEM observations were also performed to determine the crystal structure of the martensite phase. The selected area diffraction patterns (SADPs) of the aged sample at 290 and 81 K are shown in Figure 3b,c. The incident electron beam was parallel to the [001] direction of the parent phase. The fundamental spots of the typical BCC structure and additional 200 superlattice reflections are seen at 290 K. Upon cooling to 81 K, as shown in Figure 3c, four extra spots are seen among the fundamental spots, which suggest a ten‐layered crystal structure; the XRD patterns are in good agreement with the 10*M* structure. The symmetry of the martensite phase is monoclinic because the peaks of 2 0 10 and 2 0 $\bar{10}$ split in the XRD pattern, and deviation from the right angle is seen in the SADP. Thus, a martensite phase with a monoclinic 10*M* crystal structure can be inferred, which has also been confirmed for Ni–Mn–Ga Heusler alloys.^[^ ^34^, ^35^, ^36^ ^]^ In this study, we focus on the total lattice distortion of the phase transformation and do not consider precise atomic positions. The fitted lattice parameters of the 10*M* structure in the XRD patterns were $a_{10M}$ = 4.374 Å, $b_{10M}$ = 6.116 Å, $c_{10M}$ = 21.82 Å, and β = 90.2° at 90 K, and the determined β angle was in good agreement with the SADP. Recently, density functional theory studies suggested the possibility of martensitic transformation from *L*2_1_ to a tetragonal structure with *c*/*a* ≈ 1.3 in Pd_2_MnGa;^[^ ^37^, ^38^, ^39^ ^]^ however, a different martensite phase was confirmed experimentally.

**Figure 4:**
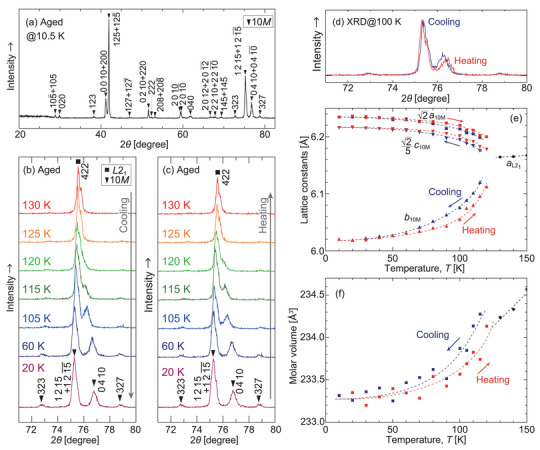
a) X‐ray diffraction (XRD) patterns of the aged Pd_2_MnGa at 10.5 K. All the peaks seen were indexed as the 10*M* structure. b,c) In situ powder XRD patterns of the aged Pd_2_MnGa alloy from 10.5 to 130 K during cooling (b) and heating (c). Below 120 K, the peaks corresponding to the martensite phase were clearly observed, and their positions shifted by changing the temperature. d) Lattice constants determined from 10.5 to 150 K: $\sqrt{2}a_{10M}$, $b_{10M}$, and $\sqrt{2}/5c_{10M}$ are plotted for comparison with $a_{L2_{1}}$. The hysteresis in the lattice constants of the martensite phase should be noted. The dashed lines are observation guides. e) Comparison of the XRD patterns at the same temperature of 100 K during heating and cooling. f) Molar volume calculated from lattice constants.

In situ XRD measurements were performed at various temperatures down to 10.5 K. The XRD pattern of the aged Pd_2_MnGa alloy at 10.5 K is shown in **Figure** **4**a. All the peaks were indexed as the 10*M* structure, and the crystal structure did not change during cooling. The XRD patterns in the range of 71° ⩽ 2*θ* ⩽ 80° for cooling and heating are summarized in Figure 4b, c, respectively. The reversibility of the martensitic transformation was also confirmed by in situ XRD measurements. The lattice constants were determined for each temperature, and the results are summarized in Figure 4e. $\sqrt{2}a_{10M}$, $b_{10M}$, and $\sqrt{2}/5c_{10M}$ are plotted for comparison with $a_{L2_{1}}$ because the β angle is almost vertical (β ≈ 90.2°). The lattice constants of the martensite phase vary gradually with variations in temperature.

Interestingly, hysteresis exists for the cooling and heating processes in the lattice constants of the martensite phase. A comparison of the XRD patterns at 100 K during heating and cooling is shown in Figure 4d, where a clear difference in the angles of the peaks is confirmed. The exact origin of the hysteresis of the lattice constants is not understood; however, the magnetization difference between cooling and heating in the martensite phase, shown in Figure 1a, may be related to this phenomenon. The molar volumes calculated from the lattice constants are depicted in Figure 4f. The volume change due to the martensitic transformation was found small.

### 2.3 Magnetic‐Field‐Induced Phase Transformation

**Figure 5:**
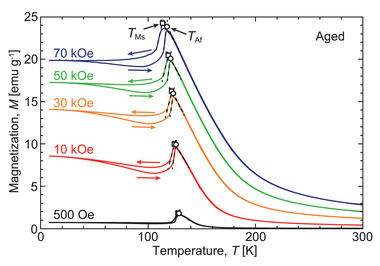
Thermomagnetization curves of the aged Pd_2_MnGa alloy under various magnetic fields. The *T*_Ms_ and *T*_Af_ were determined using extrapolation methods.

Magnetic properties were investigated under a strong magnetic field. The thermomagnetization curves under various magnetic fields up to 70 kOe are shown in **Figure** **5**. Magnetization increased, and the martensitic transformation temperatures decreased by ≈7.8 K as the magnetic field increased to 70 kOe, which is similar to the results of Ni–Mn‐based MMSMAs.^[^ ^3^ ^]^

**Figure 6:**
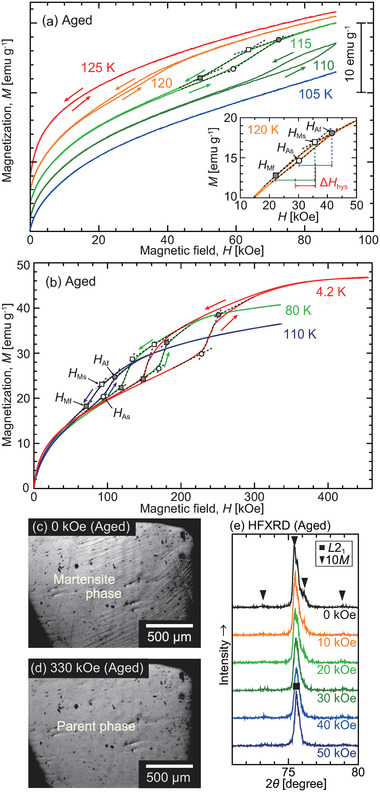
Magnetization curves for the aged Pd_2_MnGa alloy under a) relatively lower magnetic fields obtained using the physical properties measurement system–AC measurement system (PPMS‐ACMS) and b) strong magnetic fields obtained using pulsed magnets. Hysteresis are seen in the magnetization curves. c,d) In situ optical microscopy images under a pulsed magnetic field at 10 K. The surface relief disappeared with the application of the magnetic field. e) High‐field X‐ray diffraction (HFXRD) patterns of the aged powder sample under magnetic fields up to 50 kOe at 119 K. The peaks of the martensite phase changed to that of the parent phase when the magnetic field was applied. These results confirmed a magnetic‐field‐induced reverse martensitic transformation in the Pd_2_MnGa alloy for the first time. The critical magnetic fields for the phase transformation (*H*_Ms_ and *H*_Mf_) were determined by extrapolating the magnetization curves (a,b).

Magnetization measurements were performed for the aged Pd_2_MnGa alloy at various temperatures. The magnetization curves obtained using the AC measurement system (ACMS) option of the physical properties measurement system (PPMS; Quantum Design Inc., USA) and strong pulsed magnetic fields are shown in **Figures** **6**a,b, respectively. Hysteresis was observed in the magnetization curves below 120 K, which suggests a magnetic‐field‐induced transition.

To confirm the transformation, in situ optical microscopy (OM) observation was performed in high‐pulsed magnetic fields to directly see the variation in the microstructure (Video S1, Supporting Information). The OM images captured under magnetic fields of 0 and 330 kOe at 10 K are shown in Figure 6c,d, respectively, where the disappearance of the surface relief during the reverse martensitic transformation can be observed when the magnetic field is applied. Further, variations in the crystal structure were directly investigated using high‐field XRD (HFXRD) measurements, as shown in Figure 6e. The powdered sample was cooled to 10.5 K without a magnetic field, and measurements under magnetic fields were performed after heating the sample to 119 K. The sample was in the martensite phase at 119 K under 0 kOe, and the peaks indicating the martensite phase changed to those indicating the parent phase when the magnetic field was applied. Based on the results of the magnetization measurements, microstructure observations, and XRD measurements, the magnetic‐field‐induced reverse martensitic transformation was confirmed in a Pd_2_MnGa alloy for the first time.

**Figure 7:**
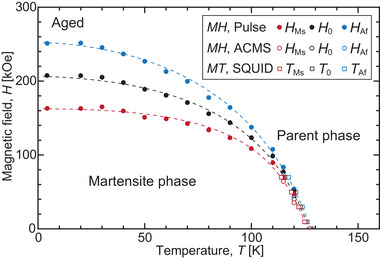
Magnetic phase diagram of the aged Pd_2_MnGa alloy determined through thermomagnetization and magnetization measurements. The dashed lines are observation guides.

The critical magnetic fields of *H*_Ms_ (forward martensitic transformation starting magnetic field) and *H*_Af_ (reverse martensitic transformation finishing field) were determined by the extrapolation method, as shown in Figure 6a,b. **Figure** **7** shows the magnetic phase diagram constructed from the thermomagnetization and magnetization measurements. The equilibrium temperature ($T_{0}=\left(T_{\mathrm{Ms}}+T_{\mathrm{Af}}\right)/2$) and magnetic field ($H_{0}=\left(H_{\mathrm{Ms}}+H_{\mathrm{Af}}\right)/2$) are defined. The relationship between *H*_0_ and temperature is described using the Clausius–Clapeyron equation

(1) $$\frac{dH_{0}}{dT}=-\frac{\Delta S}{\Delta M}$$

where Δ*M* (= *M*_M_ − *M*_P_) and Δ*S* (= *S*_M_ − *S*_P_) represent the differences in the magnetization and entropy between the parent (P) and martensite (M) phases, respectively. Δ*S* was estimated to be −0.03 J mol^−1^ K^−1^ at 120 K using Equation (1) with d*H*_0_/d*T* = −5.5 kOe K^−1^ (Figure 7) and Δ*M* = −0.6 emu g^−1^ (Figure 6a). It should be noted that the shift in the transformation temperature against the magnetic field (d*T*_0_/d*H*) corresponds well with the shift in the critical magnetic field against the changing temperature (d*H*_0_/d*T*), as can be seen in Figure 7. The value of d*T*_0_/d*H*(= −0.18 K kOe^−1^) in the Pd_2_MnGa alloy is lower than those in Ni–Mn‐based MMSMAs, that is, usually approximately −0.6 K kOe^−1^.^[^ ^19^ ^]^ An increase in Δ*M* may be effective in improving the response against the magnetic field.

**Figure 8:**
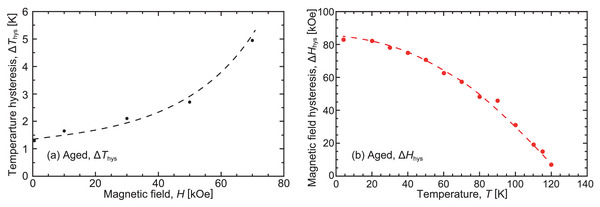
a) Δ*T*_hys_ plotted against the applied magnetic field. b) Temperature dependence of Δ*H*_hys_ for the magnetic‐field‐induced phase transformation. Δ*H*_hys_ increases at low temperatures.

The values of Δ*T*_hys_ at the various applied magnetic fields are depicted in **Figure** **8**a. The Δ*T*_hys_ increased with an increase in the magnetic field. The magnetic‐field hysteresis (Δ*H*_hys_) was also evaluated using $\Delta H_{\mathrm{hys}}=H_{A0}-H_{M0}$, where $H_{A0}=\left(H_{\mathrm{As}}+H_{\mathrm{Af}}\right)/2$ and $H_{M0}=\left(H_{\mathrm{Ms}}+H_{\mathrm{Mf}}\right)/2$, respectively. The results are plotted in Figure 8b. A small magnetic‐field hysteresis (Δ*H*_hys_) (=7 kOe only) was confirmed at 120 K, and Δ*H*_hys_ increased with a decrease in the temperature.

### 2.4 Magnetic‐Field‐Induced Strain

**Figure 9:**
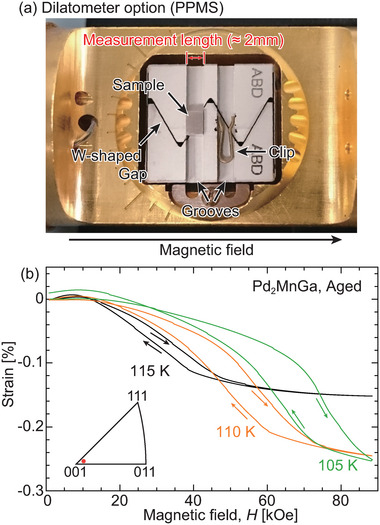
Magnetic‐field‐induced strain measurements for the aged single‐crystal Pd_2_MnGa alloy using a dilatometer equipped with a PPMS. a) Photograph of the dilatometer. b) Results of magnetic‐field‐induced strain measurements for a single crystal of the aged Pd_2_MnGa alloy.

The magnetic‐field‐induced strain was investigated using a dilatometer equipped with a PPMS. The setup is illustrated in **Figure** **9**a, where the sample was placed on one side of the groove and a clip was placed on the other side to hold the sample. When the length of the sample changed under the application of a magnetic field, the width of the W‐shaped gap changed. This width was measured using the capacitance method, from which the length of the sample was evaluated. The sample holder was rotated such that the measurement direction was parallel to the applied magnetic field.

A single crystal of the aged Pd_2_MnGa alloy was used for these measurements under magnetic fields up to 89 kOe. The crystal orientation along the direction of the magnetic field (that is, the direction of the magnetic‐field‐induced strain) is indicated in the inset of Figure 9b, which is close to the [001] direction. The measurements of the second cycle at each temperature are shown in Figure 9b. The test temperatures were 105, 110, and 115 K, which were slightly lower than *T*_Mf_ under 500 Oe, as shown in Figure 5. Moreover, the reverse transformation is expected to occur under a high magnetic field at these temperatures. Reversible magnetic‐field‐induced strains of up to 0.26% were recorded for the Pd_2_MnGa alloy. The strains measured under the application of a magnetic field are caused by the reverse martensitic transformation, and the reversible strain recorded by removing the field indicates that the same martensite variants were formed.

In this study, the measurements of the magnetic‐field‐induced strain were performed without any pre‐strains. Therefore, the measured magnetic‐field‐induced strains are indicative of a phase transformation between the multivariant martensite and parent phases. As depicted in Figure S1, Supporting Information, the maximum transformation strain was estimated as 0.76% using the phenomenological theory of martensitic transformations.^[^ ^40^, ^41^ ^]^ This value is smaller than those of Ni–Mn‐based MMSMAs (more than 6%)^[^ ^4^ ^]^ but larger than those of typical magnetostriction materials such as Tb–Dy–Fe (Terfenol‐D) alloys, which are reported to be ≈0.2–0.4%.^[^ ^42^, ^43^ ^]^ The measurement of magnetic‐field‐induced strain on a single‐variant‐martensite state will be carried out in future work. Furthermore, the large transformation width for the full martensitic transformation (≈40 kOe at 115 K) should be reduced to suppress the required magnetic fields, and an adjustment of the martensitic transformation temperatures is also necessary if the material is to be used near room temperature.

## 3 Discussion

### 3.1 Lattice Compatibility and Δ*T*_hys_

Recently, Cui et al. investigated Ti–Ni‐based alloys and related the hysteresis of the martensitic transformation to the middle eigenvalue (λ_2_) of the transformation stretch matrix (**U**), which represents the lattice compatibility of the martensitic transformation.^[^ ^16^ ^]^ A similar investigation was carried out for the Δ*T*_hys_ value of Ni–Mn‐based alloys, and the tendency of Δ*T*_hys_ to decrease was confirmed when λ_2_ approaches unity.^[^ ^19^ ^]^ According to James et al., there are 12 transformation stretch matrices for the cubic‐to‐monoclinic martensitic transformation; the following matrix was applied to the Pd_2_MnGa alloy^[^ ^17^ ^]^

(2) $$U=\left(\begin{array}{ccc} 1.0022 & 0 & -0.0018\\ 0 & 0.9916 & 0\\ -0.0018 & 0 & 1.0057 \end{array}\right).$$

**Figure 10:**
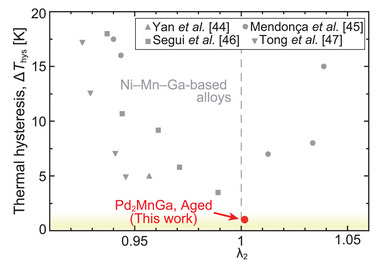
Comparison of the λ_2_ value of the aged Pd_2_MnGa alloy with those of Ni–Mn–Ga‐based alloys.^[^ ^44^, ^45^, ^46^, ^47^ ^]^

The following lattice constants were used for the calculations: *a*_10M_ = 4.384 Å, *b*_10M_ = 6.112 Å, *c*_10M_ = 21.84 Å, and β = 90.2° at 120 K for heating, and $a_{L2_{1}}$ = 6.164 Å at 130 K. The value of λ_2_ was calculated to be 1.0015, and this value was compared with those of other reported Ni–Mn–Ga‐based alloys, as shown in **Figure** **10**.^[^ ^44^, ^45^, ^46^, ^47^ ^]^ The λ_2_ value of the Pd_2_MnGa alloy was seen to be very close to unity. Thus, it is concluded that the good lattice compatibility of the martensitic transformation is one of the possible reasons for low hysteresis in Pd_2_MnGa alloys.

For this alloy, both Δ*T*_hys_ and Δ*H*_hys_ increase as the temperature decreases, as depicted in Figures 7 and 8b. In addition, the value of Δ*M* of the metamagnetic transition also increases with a decrease in the temperature, as depicted in Figure 6a,b. A similar phenomenon has also been confirmed in Ni–Mn‐based MMSMAs, and the temperature dependence of Δ*H*_hys_ was fitted based on a phenomenological model describing the kinetics of the martensitic transformation.^[^ ^48^ ^]^ However, the temperature dependence of hysteresis for the Pd_2_MnGa alloy could not be explained using this model. For this alloy, the lattice constants vary significantly with temperature (Figure 4e); thus the value of λ_2_, which affects hysteresis, is also expected to vary significantly. Further investigation of the lattice constants in the parent and martensite phases under high magnetic fields is required, which will be taken up as future work.

### 3.2 Comparison of *E*_dis_ and Δ*H*_hys_

In this section, we further discuss the value of *E*_dis_, which represents the energy loss during the martensitic transformation. The *E*_dis_ value for the magnetic‐field‐induced martensitic transformation ($E_{\mathrm{dis}}^{M-H}$) can be calculated through integration over the hysteresis loop of the magnetization curve, as follows

(3) $$E_{\mathrm{dis}}^{M-H}=-\left(\int_{0}^{H_{1}}M_{\mathrm{inc}}dH+\int_{H_{1}}^{0}M_{\mathrm{dec}}dH\right)$$

where *M*_inc_ and *M*_dec_ represent the magnetizations during the processes of increasing and decreasing the magnetic fields to *H*_1_, respectively. The *E*_dis_ of the Pd_2_MnGa alloy was estimated using Equation (3). This equation can be further approximated as follows

(4) $$E_{\mathrm{dis}}^{M-H}\approx \Delta M\cdot \Delta H_{\mathrm{hys}}$$

where Δ*M* is the difference in magnetization between the parent and martensite phases. Thus, a low value of *E*_dis_ is also beneficial for obtaining a low value of Δ*H*_hys_. A large *E*_dis_ is beneficial for use in energy‐absorption materials such as dampers.^[^ ^49^, ^50^ ^]^ However, for most applications, such as actuators, magnetocaloric refrigerants, and sensors, a small value of *E*_dis_ is favored owing to the reduced energy loss and decreased hysteresis.

**Figure 11:**
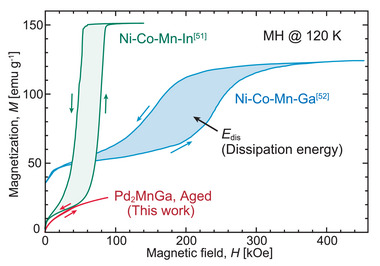
Comparison of the magnetization curves of Pd_2_MnGa, Ni–Co–Mn–In^[^ ^51^ ^]^, and Ni–Co–Mn–Ga^[^ ^52^ ^]^ alloys at 120 K. The colored areas of the hysteresis loops correspond to the dissipation energy (*E*_dis_); the *E*_dis_ of Pd_2_MnGa is the smallest.

**Figure** **11** shows a comparison of the magnetization curve of the aged Pd_2_MnGa alloy with those of conventional MMSMAs at 120 K.^[^ ^51^, ^52^ ^]^ The colored areas in the magnetization curves correspond to *E*_dis_. It can be seen that the *E*_dis_ of Pd_2_MnGa alloy is much smaller than those of Ni–Co–Mn–In and Ni–Co–Mn–Ga.

**Figure 12:**
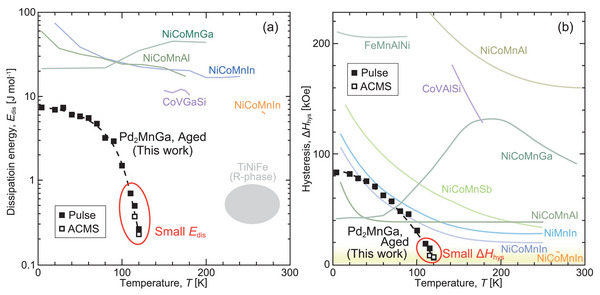
Comparison of a) *E*_dis_ and b) Δ*H*_hys_ for metamagnetic shape memory alloys (MMSMAs). The *E*_dis_ value was estimated from the areas of the hysteresis loops in either *MH* or the stress–strain curves. The *E*_dis_ and Δ*H*_hys_ values of the aged Pd_2_MnGa alloy are the smallest when compared with those of other MMSMAs. The dashed lines are observation guides.^[^ ^15^, ^19^, ^23^, ^51^, ^52^, ^53^, ^54^, ^55^ ^]^

A comparison of the temperature dependence of the *E*_dis_ value for the aged Pd_2_MnGa alloy with those of other MMSMAs is presented in **Figure** **12**a.^[^ ^15^, ^51^, ^52^, ^53^, ^54^, ^55^ ^]^ Some alloys such as La–Fe–Si,^[^ ^56^ ^]^ Mn–Co–Ge,^[^ ^57^ ^]^ and Mn_3_ZnInN^[^ ^58^ ^]^ also exhibit metamagnetic transitions with small magnetic‐field hysteresis. However, these materials are not included in this comparison because their transitions are not accompanied by variations in the crystal structures; thus, these materials are not categorized as MMSMAs. In this study, the *E*_dis_ of Pd_2_MnGa is only ≈0.3 J mol^−1^ at 120 K, which is extremely small when compared with other MMSMAs. One possible reason for the small *E*_dis_ is the good lattice compatibility of the martensitic transformation, as exhibited in Figure 10. Furthermore, at temperatures below 120 K, the aged Pd_2_MnGa alloy has the smallest *E*_dis_ among the reported MMSMAs.

In the cases of thermal or stress‐induced martensitic transformations, low values of *E*_dis_ have been confirmed in a few materials, such as the *B*2‐R phase transformation in Ti–Ni alloys (0.3–1.0 J mol^−1^) and the FCC‐FCT transformation in Fe–Pd alloys (0.3 J mol^−1^).^[^ ^22^, ^23^ ^]^ Some alloys, such as Fe–Pd, Ti–Ni, and Ni–Co–Fe–Ga, also exhibit superelasticity with narrow hysteresis;^[^ ^22^, ^23^, ^59^, ^60^ ^]^ however, these alloys cannot be driven by magnetic fields. When limited to MMSMAs, the value of *E*_dis_ is generally as high as 5–150 J mol^−1^,^[^ ^19^, ^21^, ^61^ ^]^ and the realization of a low value of *E*_dis_ is difficult; thus, the proposed Pd_2_MnGa alloy is a breakthrough in this family of alloys.

A comparison of the Δ*H*_hys_ value with those of other MMSMAs is presented in Figure 12b. For MMSMAs, a low value of Δ*H*_hys_ is necessary for applications because the creation of a strong magnetic field is energy‐consuming. Several investigations have been conducted to reduce Δ*H*_hys_, particularly for Ni–Mn‐based alloys. The suppression of Δ*H*_hys_ (11 kOe) was successfully demonstrated for the Ni–Co–Mn–In alloy at approximately room temperature;^[^ ^19^ ^]^ however, this value is still higher than 10 kOe. Moreover, obtaining a small hysteresis at low temperatures for Ni–Mn‐based alloys is more difficult because Δ*H*_hys_ increases at low temperatures.^[^ ^15^, ^51^ ^]^ The proposed Pd_2_MnGa alloy exhibits a small hysteresis of 7 kOe at 120 K, which is smaller than that of any known Ni–Mn‐based MMSMA.

## 4 Conclusions

This study reports for the first time a new Pd_2_MnGa MMSMA with extremely low dissipation energy and hysteresis. The microstructure, crystal structure, magnetic properties, martensitic transformation behavior, and magnetic‐field‐induced strain were investigated, and the results are summarized as follows.

The Pd_2_MnGa alloy ordered from the *B*2 to *L*2_1_ structure through an aging heat treatment at 573 K. The aged alloy exhibited a thermoelastic martensitic transformation from the *L*2_1_ to 10*M* structure at 127.4 K with an extremely small Δ*T*_hys_ of 1.3 K. The martensitic transformation exhibited a difference in magnetization between the parent and martensite phases, and a magnetic‐field‐induced reverse martensitic transformation was confirmed. The values of *E*_dis_ and Δ*H*_hys_ were extremely low: 0.3 J mol^−1^ and 7 kOe, respectively. The low *E*_dis_ and hysteresis values can be explained by the good lattice compatibility of the martensitic transformation with λ_2_ = 1.0015, which is very close to 1. For a single‐crystal sample, a maximum magnetic‐field‐induced strain of −0.26% was obtained at 105 K through the magnetic‐field‐induced phase transformation.

The small Δ*T*_hys_, Δ*H*_hys_, and *E*_dis_ are unique characteristics of the Pd_2_MnGa MMSMA. This alloy has the potential to be used as a low‐temperature actuator, which is an essential technology for meeting the demands of both the aerospace industry and the upcoming hydrogen society.

## 5 Experimental Section

The Pd_2_MnGa alloys were prepared by repeated arc melting or induction melting in an argon atmosphere from pure Pd (99.9%), Mn (99.9%), and Ga (99.9999%) metals. The prepared samples were sealed in quartz tubes in an argon atmosphere for an SHT at 1473 K from 24 to 72 h and then quenched in ice water after the quartz tubes were broken. The samples were bounded by tungsten wires to prevent contact with the quartz tubes. The quenched samples were further aged at 573 K for 72 h in sealed quartz tubes under an argon atmosphere. The SHT and aged samples were cut into small pieces, and the experiments described below were performed.

The thermomagnetization curves were measured using a superconducting quantum interference device (SQUID) magnetometer (Quantum Design Inc., USA) at 2 K min^−1^ under applied magnetic fields of up to 70 kOe. Magnetization measurements up to 89 kOe were performed using the ACMS equipped with PPMS at a rate of 50 Oe s^−1^. The magnetization measurements and microstructure observations using an in situ optical microscope (OM) in high‐pulsed magnetic fields were carried out at the Institute for Solid State Physics, The University of Tokyo. The maximum magnetic field and duration of the pulsed magnetic fields were ≈550 kOe and 36 ms for the magnetization measurements, respectively, and 330 kOe and 4.6 ms for the in situ OM observations, respectively. Details of the experimental methods are described in the literature.^[^ ^62^, ^63^ ^]^

The crystal structures and lattice constants at low temperatures under magnetic fields were determined through HFXRD measurements using powdered samples.^[^ ^64^ ^]^ The SHT sample was crushed into powders with grain sizes from 65 to 150 µm, sealed in quartz tubes in an argon atmosphere, and heat‐treated at 1473 K for 90 s to relieve the strain introduced during crushing. After the strain‐relief heat treatment, the powders were quenched in ice water without breaking the quartz tube. The quenched sample was aged at 573 K for 72 h before the XRD measurements. The chemical composition of the aged powders was analyzed by an electron probe microanalyzer equipped with wavelength dispersive X‐ray spectroscopes. The analyzed composition (Pd_50.29 ± 0.05_Mn_24.29 ± 0.06_Ga_25.42 ± 0.04_) is consistent with that of the bulk sample (Pd_50.26 ± 0.04_Mn_24.34 ± 0.07_Ga_25.40 ± 0.08_). The HFXRD measurements were performed at 10 ⩽ *T* ⩽ 300 K and 0 ⩽ *H* ⩽ 50 kOe.

TEM was used for the microstructural observation and crystal structure determination. Disk‐shaped samples were cut using an electrical discharge machine and then wet‐polished to a thickness of 50–100 µm. Subsequently, jet polishing was performed using an electrolyte comprising 6% perchloric acid, 12% acetic acid, 12% ethylene glycol, and 70% ethanol. Atomic‐resolution HAADF‐STEM images were obtained using an electron microscope (JEM‐ARM200F, JEOL Ltd., Japan). In situ TEM observations were performed with an electron microscope (JEM‐2100 HC, JEOL Ltd., Japan) using a sample‐cooling holder. The acceleration voltage used for the observations was 200 kV.

The magnetic‐field‐induced strain was measured using a dilatometer equipped with PPMS. A cuboid‐shaped single crystal was cut using an electron discharge machine from the large grain of the aged sample, and the oxide layer formed during processing was removed by wet polishing. The sample size was ≈2 mm × 2.5 mm × 3 mm, and the length of the 2 mm side was measured using a dilatometer. The crystal orientation of the sample was determined using electron backscattered diffraction. A magnetic field of up to 89 kOe was applied parallel to the direction of measurement, and the magnetic‐field‐induced strain was investigated. The dilatometer is described in Section 2.4 (Figure 9a).

### Statistical Analysis

For the measurements of chemical compositions of bulk and powdered Pd_2_MnGa samples using an electron probe microanalyzer, seven analyses were conducted for each sample. The average of the seven data points for each sample was taken as the determined composition with the standard error. For the magnetization measurements using high‐pulsed magnetic fields up to ≈550 kOe, time derivatives of magnetic flux density (dμ_0_ *H*/d*t*) and magnetization (d*M*/d*t*) were directly obtained by induced voltages in pick‐up coils at the desired temperature. Since d*M*/d*t* contains signals from background and uncompensated induced voltage, two measurements, with the specimen and the background, that is, sample holder, were conducted; their difference is the net d*M*/d*t* signal from the specimen. Both dμ_0_ *H*/d*t* and subtracted d*M*/d*t* were integrated with respect to time, and the preliminary *M*–*H* data were obtained. The absolute value of magnetic flux density was calibrated using the metamagnetic transition fields of MnF_2_. Thus, obtained preliminary values of *M*, *M*_preliminary_, were subjected to a calibration. Using *M*_calibrated_(*H*) = *aM*_preliminary_(*H*) + *bH* + *c*, calibration was performed by determining the coefficients *a*, *b*, and *c* in comparison with the absolute values of *M*, *M*_calibrated_, up to 70 kOe obtained by a SQUID magnetometer at the same temperature. Since other data were directly obtained, neither data processing nor statistic procedures were applied.

## Conflict of Interest

The authors declare no conflict of interest.

## Supporting information

Supporting Information

Supplemental Movie 1

## Data Availability

The data that support the findings of this study are available from the corresponding author upon reasonable request.
