# Supplementary material for: Pd2MnGa Metamagnetic Shape Memory Alloy with Small Energy Loss
Source: Adv Sci (Weinh). 2023 Jun 12;10(23):2207779. doi: 10.1002/advs.202207779 (PMC10427369; doi:10.1002/advs.202207779)
Supplement: Supplementary file 1 — Supporting Information [file ADVS-10-2207779-s002.pdf]

ADVANCED SCIENCE
Open Access

Supporting Information

for *Adv. Sci.*, DOI 10.1002/advs.202207779

Pd_2MnGa Metamagnetic Shape Memory Alloy with Small Energy Loss

Tatsuya Ito, Xiao Xu, Atsushi Miyake, Yuto Kinoshita, Makoto Nagasako, Kohki Takahashi, Toshihiro Omori, Masashi Tokunaga and Ryosuke Kainuma*

Supporting Information

Pd_2MnGa metamagnetic shape memory alloy with small energy loss

Tatsuya Ito[1], *Xiao Xu*[1,2]*, *Atsushi Miyake*[3], *Yuto Kinoshita*[3], *Makoto Nagasako*[4], *Kohki Takahashi*[4], *Toshihiro Omori*[1], *Masashi Tokunaga*[3], *Ryosuke Kainuma*[4]

[1]*Department of Materials Science, Graduate School of Engineering, Tohoku University, Aobayama 6-6-02, Sendai 980-8579, Japan*

[2] *Organization for Advanced Studies, Tohoku University, Sendai 980-8577, Japan*

[3] *The Institute for Solid State Physics, The University of Tokyo, Kashiwa 277-8581, Japan*

[4] *Institute for Materials Research, Tohoku University, Sendai 980-8577, Japan*

Corresponding author: Email: xu@material.tohoku.ac.jp

Supporting Discussion

Calculation of the transformation strain

The transformation strain in the single variant state was calculated from the determined lattice constants, and the result is shown in Figure S1. The estimated maximum transformation strain was 0.76% for the <001> direction.

Supporting Figure

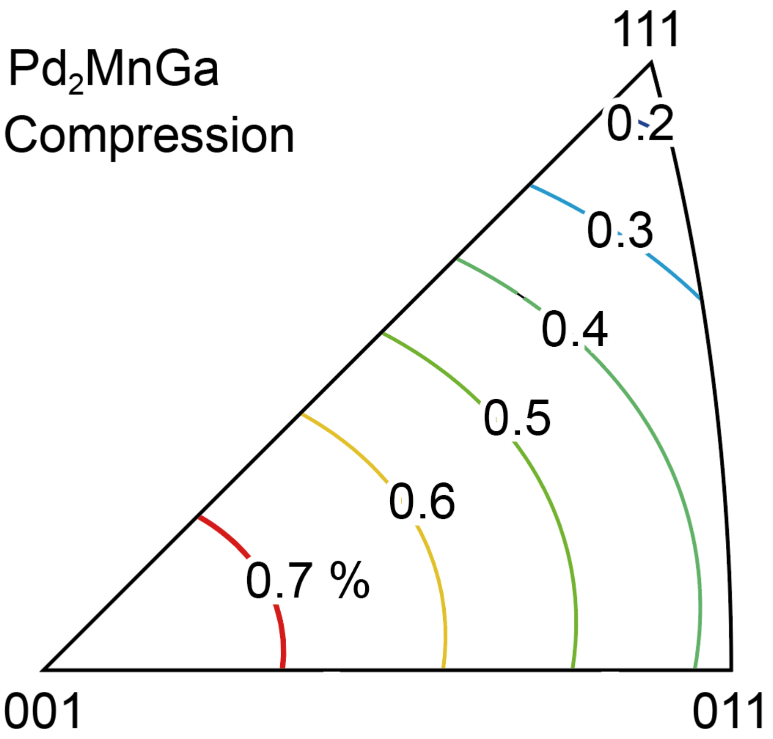

Figure S1: Calculated transformation strain by compression based on the shape strain of martensitic transformation using the experimental lattice constants.

Supporting Video

Video S1: Video showing the change of microstructure by in situ optical microscopy observation in a high-pulsed magnetic field. The sample was at 10 K, and a magnetic field up to 330 kOe was applied. The disappearance of the surface relief is direct evidence of the magnetic-field-induced reverse martensitic transformation.
